# The right ventricular dysfunction and ventricular interdependence in patients with DM: assessment using cardiac MR feature tracking

**DOI:** 10.1186/s12933-023-01806-7

**Published:** 2023-04-21

**Authors:** Rui Shi, Zhi-Gang Yang, Ying-Kun Guo, Wen-Lei Qian, Yue Gao, Xue-Ming Li, Li Jiang, Hua-Yan Xu, Yuan Li

**Affiliations:** 1 Department of Radiology, West China Hospital, Sichuan University, No. 37# Guo Xue Xiang, Chengdu, 610041 Sichuan China; 2 Department of Radiology, Key Laboratory of Birth Defects and Related Diseases of Women and Children of Ministry of Education, West China Second University Hospital, Sichuan University, Chengdu, China

**Keywords:** Diabetes mellitus, Right ventricular dysfunction, Ventricular interdependence, CMR feature-tracking

## Abstract

**Background:**

To investigate the difference of right ventricular (RV) structural and functional alteration in patients with diabetes mellitus (DM) with preserved left ventricular ejection fraction (LVEF), and the ventricular interdependence in these patients, using cardiac MR (CMR) feature tracking.

**Methods:**

From December 2016 to February 2022, 148 clinically diagnosed patients with DM who underwent cardiac MR (CMR) in our hospital were consecutively recruited. Fifty-four healthy individuals were included as normal controls. Biventricular strains, including left/right ventricular global longitudinal strain (LV-/RVGLS), left/right ventricular global circumferential strain (LV-/RVGCS), left/right ventricular global radial strain (LV-/RVGRS) were evaluated, and compared between patients with DM and healthy controls. Multiple linear regression and mediation analyses were used to evaluate DM's direct and indirect effects on RV strains.

**Results:**

No differences were found in age (56.98 ± 10.98 vs. 57.37 ± 8.41, p = 0.985), sex (53.4% vs. 48.1%, p = 0.715), and body surface area (BSA) (1.70 ± 0.21 vs. 1.69 ± 0.17, p = 0.472) between DM and normal controls. Patients with DM had decreased RVGLS (− 21.86 ± 4.14 vs. − 24.49 ± 4.47, p = 0.001), RVGCS (− 13.16 ± 3.86 vs. − 14.92 ± 3.08, p = 0.011), and no decrease was found in RVGRS (22.62 ± 8.11 vs. 23.15 ± 9.05, p = 0.743) in patients with DM compared with normal controls. The difference in RVGLS between normal controls and patients with DM was totally mediated by LVGLS (indirect effecting: 0.655, bootstrapped 95%CI 0.138–0.265). The difference in RVGCS between normal controls and DM was partly mediated by the LVGLS (indirect effecting: 0.336, bootstrapped 95%CI 0.002–0.820) and LVGCS (indirect effecting: 0.368, bootstrapped 95%CI 0.028–0.855).

**Conclusions:**

In the patients with DM and preserved LVEF, the difference in RVGLS between DM and normal controls was totally mediated by LVGLS. Although there were partly mediating effects of LVGLS and LVGCS, the decrease in RVGCS might be directly affected by the DM.

## Introduction

Cardiovascular disease is one of the most important diabetes-related complications [1–3]. Rubler et al. identified diffused myocardial fibrosis, cardiac hypertrophy, and myocardial microangiopathy in diabetes patients and termed it diabetic cardiomyopathy [4]. Latest guidelines and the majority of studies have so far described diabetic cardiomyopathy as a disease involving the left ventricular (LV) [5–7]. It is generally accepted that diabetic cardiomyopathy presents early with left ventricular hypertrophy, increased cardiac stiffness, and impaired diastolic function and later develops into heart failure with reduced ejection fraction, which can lead to serious cardiovascular-related death [8, 9]. With the intensive study of diabetic cardiomyopathy, it has become apparent that diabetic cardiomyopathy is not a regional disease of the left ventricle but may also have a parallel effect on the right ventricular (RV) through a similar pathological process [10, 11]. It is noteworthy that the interdependence of biventricular function in diabetic cardiomyopathy cannot be ignored. Dysfunction of the left ventricular leads to reduced right ventricular function via mechanical interaction of the septal wall or pericardium [12–14]. Small studies suggest that right ventricular dysfunction may be associated with a poor prognosis for long-term cardiovascular disease [15]. Diabetes-related right ventricle dysfunction and biventricular interactions are often overlooked and, to date, remain largely unexplored.

Due to the high temporal and soft tissue resolution, CMR imaging is considered the gold standard for assessing myocardial structure and function [16]. Novel CMR postprocessing tool, such as CMR feature tracking (CMR-FT), significantly improves RV functional assessment [17, 18]. Given these considerations, we design the present study to investigate the right ventricular structural and functional characteristics in diabetic patients with preserved LV ejection fraction (LVEF) and the mediating effects of LV dysfunction on RV strain in these patients, using CMR-FT.

## Methods

### Study population

The Institutional Review Board approved this study, and informed consent was waived due to the retrospective nature of this investigation. From December 2016 to February 2022, clinically diagnosed patients with DM who underwent cardiac MR in our hospital were consecutively recruited. The inclusion criteria were:1) DM diagnosed according to the guidelines of the American Diabetes Association [19] or patients receiving glucose-lowering therapy. 2) patients with preserved LVEF on CMR (> 50%).The exclusion criteria were: (1) known cardiovascular disease, including coronary heart disease (myocardial infarction, revascularization, or coronary bypass), primary cardiomyopathy, severe valvular heart disease, congenital heart disease, and so on; (2)patients with diseases that may affect right heart function, such as chronic obstructive pulmonary disease, pulmonary artery embolism, and clinically diagnosed pulmonary hypertension; (3) incomplete critical clinical information; and (4) poor image quality cannot be used for analysis. A total of 148 patients with DM were finally recruited in this study. Fifty-four healthy individuals were included as normal controls.

Patient's demographic characteristics, clinical history, cardiovascular risk factors, and laboratory test results were recorded through Hospital Information System and Laboratory Information Management System.

### Cardiac MRI scan protocol

Cardiac MR scanning was performed using a whole-body 3.0 T Siemens MAGNETOM Trio Tim system or a MAGNETOM Skyra scanner (Siemens Medical Solutions, Erlangen, Germany). The balanced steady-state free precession cine images were obtained in standard short- and long-axis views at end-expiratory breath-hold. The parameters for the Siemens MAGNETOM Trio Tim system were: temporal resolution,40.35 ms; repetition time/echo time, 3.4/1.3 ms; matrix, 208 × 139; flip angle, 50^◦^; field of view, 250 mm × 300 mm; slice thickness, 8 mm; and the number of frames, 25 per cardiac cycle. The parameters for the MAGNETOM Skyra scanner were: temporal resolution, 39.34 ms; repetition time/ echo time 2.8/1.2 ms; flip angle, 38^◦^; slice thickness, 8 mm; the field of view, 360 mm × 300 mm; matrix size, 256 × 166; and the number of frames, 25 per cardiac cycle.

### Cardiac function and feature tracking analysis

**Fig. 1:**
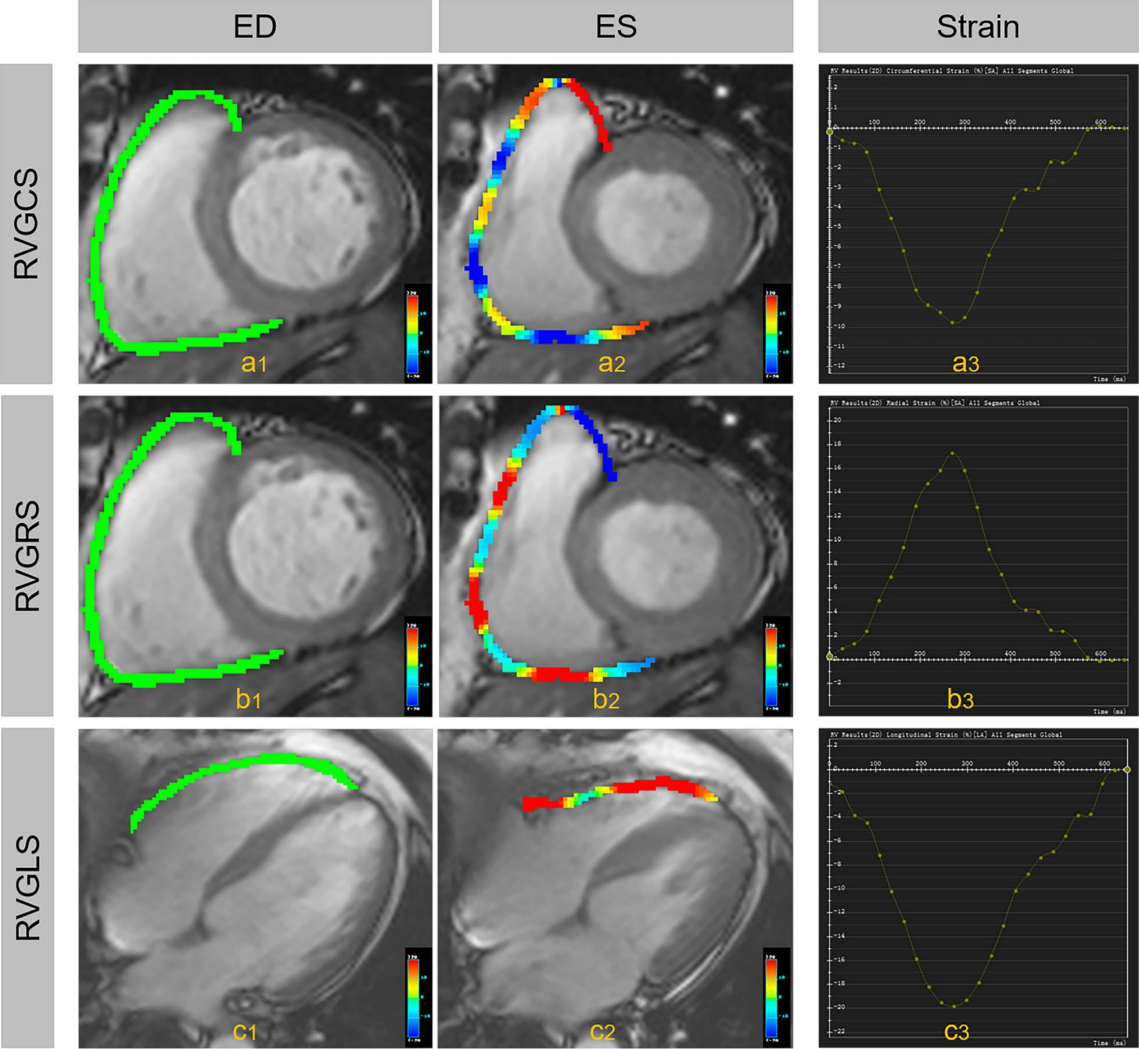
Illustration of the color-coded 2D strain for the right ventricle of a DM patient. After right ventricular epi- and endocardial borders were traced in short-axis and 4ch- longitudinal view images on end-systole (**a**2–**c**2) and end-diastole (**a**1–**c**1), RVGCS (**a**3), RVGRS (**b**3), and RVGLS (**c**3) were automatically generated. ED, end-diastolic; ES, end-systolic; RVGCS, right ventricular global circumferential strain; RVGLS, right ventricular global longitudinal strain; RVGRS, right ventricular global radial strain

All the CMR images were independently analyzed by two radiologists with more than three years of experience in CMR diagnosis using commercial software (cvi42, Circle Cardiovascular Imaging, Calgary, Canada). To calculate bi-ventricular functional parameters, including RV-/LV end-systolic volume (RV/LV ESV), RV/LV end-diastolic volume (RV/LV EDV), myocardial mass, and RV/LV ejection fraction (RV/LVEF), ventricular epi- and endocardial borders were traced in contiguous short-axis images on end-systole and end-diastole. RV/LV EDV, RV/LV ESV, and myocardial mass were standardized by body surface area (BSA). The RV/LV remodeling index (RV/LVRI) were calculated as the myocardial mass divided by the EDV. To analyze the biventricular feature tracking parameters, additional left ventricular four- and two-chamber longitudinal views and right ventricular four-chamber longitudinal views were tracked with the end-diastole set as the reference point. 3D LV strain parameters (LV global longitudinal strain (LVGLS), LV global radial strain (LVGRS), and LV global circumferential strain (LVGCS)) and 2D RV strain parameters (RV global longitudinal strain (RVGLS), RV global radial strain (RVGRS), and RV global circumferential strain (RVGCS)) were automatically generated after these contours tracing performed (Fig. 1).

### Reproducibility analysis

To determine intra- and inter-observer variability of biventricular CMR-FT parameters, 40 random subjects were measured twice by a radiologist at a 2-week interval. Another investigator reanalyzed the measurement results of the software (cvi42). The twice measurement results of the first investigators were used to assess intra-observer variability. The measurement results of the two investigators were used to assess inter-observer variability.

### Statistical analysis

Continuous variables were expressed as mean ± standard deviation (SD) or median and interquartile range. The difference between normal controls and patients with DM was tested with the Mann–Whitney U test or independent t-test. Categorical variables were expressed as frequency and percentage, and differences between groups were compared using the χ^2^ test or Fisher’s exact test. Relationships between RV strain and LV geometric parameters and strains (LVGLS, LVGCS, and LVGRS) were estimated with Pearson’s or Spearman’s correlation coefficient analysis, as appropriate.

Multivariate linear regression models adjusting for statistically significant parameters in univariate analysis (p < 0.05) and traditional clinical risk factors (age, sex, BSA, smoking history, drinking history, hypertension, and hyperlipidemia) were constructed to determine the independent effects of DM on the biventricular strains. Multicollinearity was assessed by the variance inflation factor (VIF > 10). Mediation analyses with bootstrap method were performed to assess whether ventricular interdependence statistically mediated the RV strain difference between diabetes and normal controls [20]. LV strains independently affected by diabetes (LVGLS and LVGCS) were added into the aforementioned regression models as mediators. Intraclass correlation coefficients (ICCs) were used to assess intra- and inter-observer variability of 3D LV and 2D RV strain parameters. The significance level of all analyses was set at two-side p < 0.05. The statistical analyses were performed using IBM SPSS Statistics for Windows, version 25.0 (IBM Corp., Armonk, NY, USA).

## Results

### Clinical characteristics of patients with DM

The final analysis included 148 patients with DM and preserved LVEF and 54 normal controls. The mean age of patients with DM was 56.98 ± 10.98 years, and 91 (61.5%) had hypertension. No differences were found in age (56.98 ± 10.98 vs. 57.37 ± 8.41, p = 0.985), sex (53.4% vs. 48.8%, p = 0.472), and BSA (1.70 ± 0.21 vs. 1.69 ± 0.17, p = 0.715) between DM and normal controls. The demographic and clinical characteristics of the research subjects are summarized in Table 1.

**Table 1:** Basic demographic and clinic characteristics of the study population

Characteristics	Normal controls (n = 54)	Diabetes (n = 148)	P value
Age	57.37 ± 8.41	56.98 ± 10.98	0.985
BSA	1.69 ± 0.17	1.70 ± 0.21	0.715
Sex (female), n (%)	26 (48.1)	79 (53.4)	0.472
Smoking, n (%)	–	69 (46.6)	–
Drinking, n (%)	–	28 (18.9)	–
Hypertension,n (%)	–	91 (61.5)	–
Hyperlipidemia, n (%)	–	29 (19.6)	–
Laboratory Examinations			
FBG	–	6.81 (5.52,8.31)	–
HDL-C	–	1.09 (0.89, 1.36)	–
LDL-C	–	2.33 (1.72, 2.92)	–
TG	–	1.51 (1.09, 2.17)	–
TC	–	4.16 (3.45, 4.94)	–
Medication			–
Beta-blockers	–	35 (23.6)	–
Calcium-channel blocker, n (%)	–	16 (10.8)	–
ACEI/ARB, n (%)	–	52 (35.1)	–
Diuretics, n (%)	–	26 (17.6)	–
Anti-diabetic treatment			
Insulin, n (%)	–	27 (18.2)	–
Metformin, n (%)	–	21 (14.2)	–
Sulfonylurea, n (%)	–	40 (27.0)	–
α-Glucosidase inhibitor, n (%)	–	42 (28.4)	–
Other, n (%)	–	6 (4.1)	–
Non-drug, n (%)	–	24 (16.2)	–

Data are reported as n (%) or median (interquartile range) appropriately

BSA: Body surface area; FBG: Fasting blood sugar; HDL-C: High-density lipoprotein cholesterol; LDL-C: low-density lipoprotein cholesterol; TC: Total cholesterol; TG: Total triglycerides. GFR: Glomerular filtration rate

### Characteristics of biventricular structure and function in diabetic patients

Table 2 shows the biventricular structural and functional characteristics of diabetic patients. Compared with normal controls, patients with DM had higher heart rate (HR, 78.50 ± 13.53 vs. 72.49 ± 11.23, p = 0.008) and showed increased LV mass index (LVMI) (50.61 ± 16.28 vs. 43.69 ± 8.92, p = 0.007) and LVRI (0.69 ± 0.18 vs. 0.60 ± 0.09, p = 0.004). No differences were found in LVEF (62.95 ± 6.98 vs. 64.04 ± 4.16, p = 0.097), LVEDVI (74.67 ± 17.62 vs. 73.12 ± 11.31, p = 0.294), and LVESVI (28.18 ± 10.21 vs. 26.47 ± 6.11, p = 0.078) between the two groups. No differences were found in RVEF (61.41 ± 8.04 vs. 60.47 ± 6.59, p = 0.549), RVEDVI (58.42 ± 15.52 vs. 58.70 ± 12.49, p = 0.778), RVESVI (22.71 ± 8.22 vs. 23.37 ± 7.13, p = 0.592), RVMI (14.08 ± 3.57 vs. 14.43 ± 3.22, p = 0.462), and RVRI (0.25 ± 0.05 vs. 0.25 ± 0.04, p = 0.866) between patients with DM and normal controls.

**Table 2:** Comparison of biventricular structure and left ventricular time volume parameters between diabetic patients and normal subjects

	Normal controls (n = 54)	Diabetes (n = 148)	P value
Heart rate	72.49 ± 11.23	78.50 ± 13.53	0.008
Left ventricle			
LVEF (%)	64.04 ± 4.16	62.95 ± 6.98	0.097
LVEDVI (mL/m^2^)	73.12 ± 11.31	74.96 ± 17.62	0.294
LVESVI (mL/m^2^)	26.47 ± 6.11	28.18 ± 10.21	0.078
LVMI (g/m^2^)	43.69 ± 8.92	50.61 ± 16.28	0.007
LVRI(g/mL)	0.60 ± 0.09	0.69 ± 0.18	0.004
Right ventricle			
RVEF (%)	60.47 ± 6.59	61.41 ± 8.04	0.549
RVEDVI (mL/m^2^)	58.70 ± 12.49	58.42 ± 15.52	0.778
RVESVI (mL/m^2^)	23.37 ± 7.13	22.71 ± 8.22	0.592
RVMI (g/m^2^)	14.43 ± 3.22	14.08 ± 3.57	0.462
RVRI(g/mL)	0.25 ± 0.04	0.25 ± 0.05	0.866

Data are reported as mean ± SD

LVEF: left ventricular ejection fraction; LVEDVI: left ventricular end-diastolic volume index; LVESVI: left ventricular end-systolic volume index; LVMI: left ventricular myocardial mass index; LVRI: left ventricular remodeling index; RVEF: right ventricular ejection fraction; RVEDVI: right ventricular end-diastolic volume index; RVESVI: right ventricular end-systolic volume index; RVMI: right ventricular myocardial mass index; RVRI: right ventricular remodeling index

**Fig. 2:**
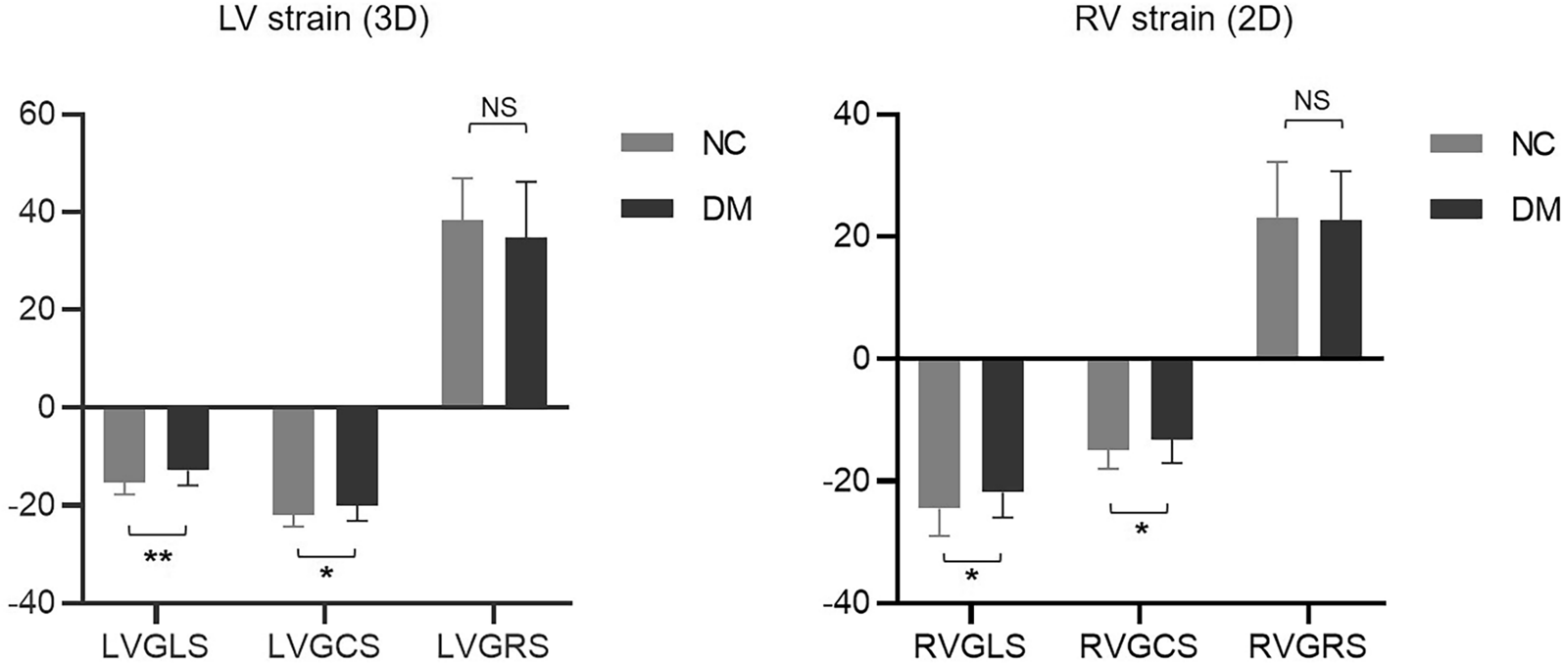
Comparing LV- and RV strains between patients with DM and normal control. LVGLS: left ventricular global longitudinal strain. LVGCS: left ventricular global circumferential strain. LVGRS: left ventricular global radial strain. NC: normal controls. DM: diabetic mellitus. Data are reported as median (25th–75th percentiles). *: p < 0.05 between DM and NC. **: p < 0.001 between DM and NC. NS: not significant

The comparison of biventricular strain between the DM and normal controls is shown in Fig. 2. Compared to normal controls, patients with DM had lower LVGLS (− 12.76 ± 3.09 vs. − 15.27 ± 2.42, p < 0.001) and LVGCS (− 19.95 ± 3.14 vs. − 21.88 ± 2.4, p = 0.001), and the LVGRS (34.67 ± 11.57 vs. 38.41 ± 8.51, p = 0.066) was not significant reduced. Compared to normal controls, patients with DM had decreased RVGLS (− 21.86 ± 4.14 vs. − 24.49 ± 4.47, p = 0.001) and RVGCS (− 13.16 ± 3.86 vs. − 14.92 ± 3.08, p = 0.011). No decrease was found in RVGRS (22.62 ± 8.11 vs. 23.15 ± 9.05, p = 0.743) in patients with DM compared with normal controls.

### Correlation between RV strains and LV structure and function

RVGLS were significantly correlated with LVEF (r = − 0.258, p = 0.001), LVEDVI (r = 0.240, p = 0.002), LVESVI (r = 0.278, p < 0.001), LVRI (r = 0.161, p = 0.040), LVMI (r = 0.328, p < 0.001), LVGLS (r = 0.334, p < 0.001), LVGCS (r = 0.378, p < 0.001), and LVGRS (− 0.310, p < 0.001). RVGCS were significantly correlated with LVEF (r = − 0.281, p = 0.001), LVEDVI (r = 0.249, p = 0.008), LVESVI (r = 0.309, p < 0.001), LVMI (0.159, p = 0.043), LVGLS (r = 0.232, p = 0.009), LVGCS (r = 0.360, p < 0.001), and LVGRS (− 0.368, p < 0.001). RVGRS were significantly correlated with LVEF (r = 0.289, p < 0.001), LVEDVI (r = − 0.207, p = 0.009), LVESVI (r = − 0.294, p < 0.001), LVGCS (r = − 0.291, p < 0.001), LVGLS (r = − 0.173, p = 0.029), and LVGRS (r = 0.365, p < 0.001). No significant correlation was found between RVGRS and LVMI (r = − 0.084, p = 0.292) or LVRI (r = 0.101, p = 0.203) (Table 3).

**Table 3:** The correlation between right ventricular strains and left ventricular diastolic and systolic function parameters

	RVGLS		RVGCS		RVGRS	
	r	p	r	p	r	p
LVEF (%)	− 0.258	0.001	− 0.281	0.001	0.289	< 0.001
LVEDVI (mL/m2)	0.240	0.002	0.249	0.001	− 0.207	0.009
LVESVI (mL/m2)	0.278	< 0.001	0.309	< 0.001	− 0.294	< 0.001
LVMI (g/m2)	0.328	< 0.001	0.159	0.043	− 0.084	0.292
LVRI (g/mL)	0.161	0.040	− 0.042	0.427	0.101	0.203
LVGLS, %	0.410	< 0.001	0.282	< 0.001	− 0.173	0.029
LVGCS, %	0.375	< 0.001	0.345	< 0.001	− 0.291	< 0.001
LVGRS, %	− 0.388	< 0.001	− 0.367	< 0.001	0.365	< 0.001

LVGLS: left ventricular global longitudinal strain. LVGCS: left ventricular global circumferential strain. LVGRS: left ventricular global radial strain. Other abbreviations are consistent with Table 2

### The impact of DM on biventricular structure and function

**Fig. 3:**
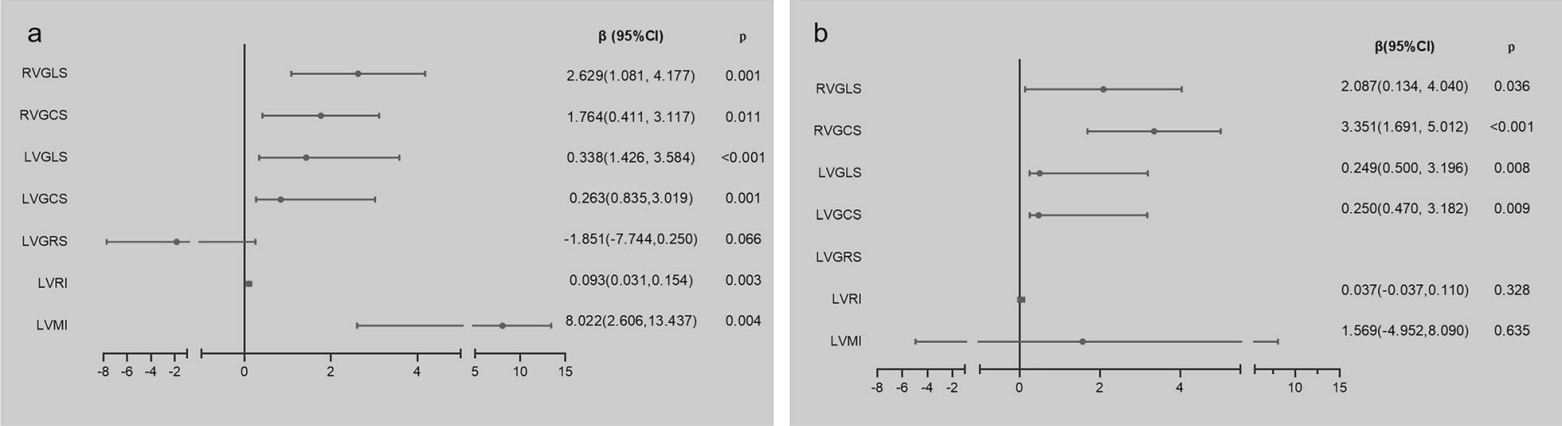
Forest plot: Univariate and Multifactorial analysis of diabetes mellitus on biventricular structural and functional parameters. Abbreviations are consistent with Table 2 and Fig. 2. Multivariable analysis corrected for age, sex, body surface area, history of smoking, history of alcohol consumption, hypertension, hyperlipidemia, FBG, HDL-C, LDL-C, and heart rate

The multivariable analysis showed that after adjusting the confounding factors (including age, sex, BSA, hypertension, hyperlipidemia, smoking, drinking, heart rate, HDL-C, LDL-C and FBG), DM was the independent impactor of LVGLS (0.249(0.500, 3.196), p = 0.008), LVGCS (0.250(0.470, 3.182), p = 0.009), RVGLS (2.087(0.134, 4.040), p = 0.036), RVGCS (3.351(1.1691, 5.012), < 0.001), but have no impact on LVRI (0.037(− 0.037,0.110), p = 0.328), LVMI (1.569(− 4.952, 8.090), p = 0.635). (Fig. 3).

### Direct and indirect effecting of DM on RV strains

Direct and indirect effects of DM on RV strains with mediators LVGLS and LVGCS are displayed in Table 4. The difference in RVGLS between normal controls and patients with DM was totally mediated by LVGLS (indirect effecting: 0.655, bootstrapped 95%CI 0.138–0.265), and LVGCS had no significant mediating effect. The difference in RVGCS between normal controls and patients with DM was partly mediated by the LVGLS (indirect effecting: 0.336, bootstrapped 95%CI 0.002–0.820) and LVGCS (indirect effecting: 0.368, bootstrapped 95%CI 0.028–0.855).

**Table 4:** Mediating effects of DM on RVGLS and RVGCS with the mediators LVGLS and LVGR

	RVGLS		RVGCS		
	Direct effect	Indirect effect	Direct effect		Indirect effect
LVGLS	1.175 (− 0.762,3.111)	0.655 (0.138,1.265)*	2.614 (0.970,4.258)*		0.336 (0.002,0.820)*
LVGCS	1.830 (− 0.123,3.783)	0.279 (− 0.088,0.905)	2.582 (0.940,4.224)*		0.368 (0.028,0.855)*

LVGLS: left ventricular global longitudinal strain; LVGCS: left ventricular global circumferential strain; RVGLS: right ventricular global longitudinal strain; RVGCS: right ventricular global circumferential strain

### Intra- and Inter-observer Reproducibility

Intra- and inter-observer variabilities of biventricular strain are shown in Table 5. The intra- and inter-observer ICCs for RV strain were between 0.672–0.874 and 0.604–0.832, for LV strain were between 0.786–0.929 and 0.734–0.845.

**Table 5:** Inter and intra-observer variability of biventricular strain

	intra-observer ICC (95%CI)	p value	inter-observer ICC (95%CI)	p value
RV strain				
GLS	0.874 (0.770, 0.932)	< 0.001	0.832 (0.699, 0.909)	< 0.001
GCS	0.672 (0.452, 0.815)	< 0.001	0.604 (0.356, 0.773)	< 0.001
GRS	0.692 (0.481, 0.827)	< 0.001	0.687 (0.473, 0.824)	< 0.001
LV strain				
GLS	0.929 (0.867, 0.962)	< 0.001	0.845 (0.701, 0.919)	< 0.001
GRS	0.786 (0.625, 0.883)	< 0.001	0.734 (0.544, 0.852)	< 0.001
GCS	0.852 (0.734, 0.920)	< 0.001	0.774 (0.606, 0.876)	< 0.001

ICC: Intraclass correlation coefficient; other abbreviations are consistent with Table 2

## Discussion

The present study investigated the RV structure and function difference between DM individuals with preserved LVEF and normal controls using CMR tissue-tracking techniques. Notwithstanding, ventricular interdependence via the septum cannot be ignored in the study of right ventricular dysfunction. We further explored the differences in RV function between DM and normal populations with LV strain as mediators. Our data revealed that RVGLS, RVGCS, LVGLS, and LVGCS were decreased in patients with DM compared with normal controls, and DM was an independent influence on the aforementioned biventricular strain. However, the difference in RVGLS between DM and normal controls was totally mediated by LVGLS. LVGLS and LVGCS partially mediated the difference in RVGCS between DM and normal controls.

### Potential pathophysiological mechanisms of diabetic cardiomyopathy

Cardiovascular disease is a common complication in patients with diabetes [8, 21]. A large number of scholars have conducted intensive research on diabetes-related cardiomyopathy. Nevertheless, the lack of a precise definition makes the study of diabetes-related cardiomyopathy pathophysiology, natural course, and associated clinical outcomes challenging. Important factors currently thought to drive the pathology of myocardial dysfunction in DM are insulin resistance/hyperinsulinemia and impaired glucose tolerance, which may be present years or more before the onset of DM. The GLP-1R is a highly effective target for managing type 2 diabetes by increasing insulin secretion, which lowers blood glucose levels. Enhanced glucose-stimulated GLP-1 responses may have positive impacts on vascular function, reducing the risk of cardiovascular disease and mortality, and lowering central and peripheral blood pressure [22]. Diabetic patients face an increased risk of developing metabolic syndrome, and in some cases, thoracic adipose tissue secretes higher levels of Cer16:0, which amplifies oxidative stress, systemic inflammation and reduces endothelium-dependent vasodilation, resulting in adverse cardiac outcomes [23]. The LV myocardial work is the main source of total cardiac work, and current studies on the pathogenesis and prognosis of diabetic cardiomyopathy focused on LV. At the early stage of diabetic cardiomyopathy onset, cardiac hypertrophy is associated with progressive impairment in LV strain and torsion, and abnormalities in left ventricular diastolic function are detected in about 75% of diabetic patients at this stage. These changes are accompanied by an upregulation of specific microRNAs targeting the extracellular matrix [24]. Extracellular volume (ECV) fraction-derived myocardial fibrosis was proven to be an independent risk factor of heart failure [25–27].

### Imaging evaluation of right ventricular dysfunction in DM

Diabetes is a systemic disease, and it is reasonable to believe that the right ventricle suffers a parallel injury. As a convenient imaging modality, most of the findings of structural and functional alterations in the RV are based on the results of ultrasound studies. The narrow acoustic window, dependence on operator interpretation, and tracking errors due to the low signal-to-noise ratio limit the use of 2D-STE. The thinner wall of the RV increases the difficulty of assessment accurately and reproducibility of 2D-STE. Longitudinal strain based on 2D speckle tracking echocardiography is promoted as an approximate of RV myocardial work [28]. Several investigators reported reduced RVGLS in diabetic patients relative to normal subjects. They omitted RVGCS into observations, considering that RVGLS was a better parameter reflecting the RV contractility [29]. In recent years, cardiologists have focused on RV geometry and function alteration in different disease spectra using CMR, which has higher tissue resolution and reproducibility. Shao et al. studied characteristics of RV function in diabetic patients with preserved LV function using CMR feature-tracking and showed impaired RVGLS in these individuals [30]. We included the patients with DM and preserved LVEF as research subjects, and the same was found that these patients had decreased RVGLS compared with normal controls. Our study further showed good reproducibility in assessing RVGCS by cardiac MR feature tracking and found reduced RVGCS in diabetic patients.

### Ventricular interdependence and the mediating effect of LV strain on RV dysfunction in patients with DM

Ventricular interdependence via the septum and limited pericardial flexibility is another possible mechanism for RV dysfunction in DC. It is theorized that LV contraction might be the primary source for RV-developed pressure, and about 20–40% of RV systolic pressure resulted from LV contraction [12]. The septum's motion and position are essential in ventricular interaction. Diffusion tensor magnetic resonance image elaborated the common myocardial fiber encircled the ventricle [31]. LV geometry and function alterations could contribute to RV dysfunction through ventricular interdependency mediated by the septum [13, 32]. LV myocardial function has been reported to be highly associated with RVGLS in diabetic patients [33–36]. The Maastricht Study found that LV structure or function indices did not statistically mediate the association between DM and RV structural changes [37].

Taken together, left ventricular strain impairment in DM is associated with RV function is well known. Whether the impact of DM on RV strains decrease is mediated by LV strains alteration remains uncertain. RV free-wall GLS decrease was totally mediated by LVGLS, whereas RVGCS impairment was partially mediated by LV function in diabetic patients with preserved LVEF. Our results were inconsistent with the study of Pauline et al. [37]. A possible explanation is a difference in the study population, as their study included patients with preserved LV function, whereas the patients with DM in our study showed significantly reduced LVGLS. This is broadly consistent with the previously proposed theory of biventricular interdependence, i.e., a weaker biventricular interaction in the presence of normal LV function [14].

### Limitations

Despite the meaningful results, several limitations of the current study cannot be ignored. Firstly, it was a single-center, retrospective study, and there might be potential selection bias. Secondly, we included diabetic patients with preserved LVEF, and the generalizability of our findings to other populations can be questioned, and whether biventricular function maintains the same interactions in DM with heart failure is worth to be further explored. Thirdly, the characteristics of retrospective studies limited our ability to consider the duration of diabetes diagnosis, it is crucial for future studies to investigate whether this variable has an impact on the changes in biventricular structure and function. Finally, the clinical outcomes, including heart failure, other cardiovascular complications, or cardiovascular death, were not available in the present study. There are available data elucidating the prognostic value of right ventricular strain [38, 39].

## Conclusions

RVGLS and RVGCS decreased in patients with DM and preserved LVEF. Abnormal diabetic metabolism could mediate reduced RVGLS mainly by impairing LVGLS in patients with preserved LVEF. Despite the partly mediating effect of LVGLS and LVGCS, the difference in RVGCS might be directly affected by the DM.

## Data Availability

The datasets generated and/or analyzed during the current study are available from the corresponding author upon reasonable request.

## Abbreviations

DM: Diabetes mellitus
CMR-FT: Feature tracking
LVEF: Left ventricular ejection fraction
LVEDVI: Left ventricular end-diastolic volume index
LVESVI: Left ventricular end-systolic volume index
LVMI: Left ventricular myocardial mass index
LVRI: Left ventricular remodeling index
RVEF: Right ventricular ejection fraction
RVEDVI: Right ventricular end-diastolic volume index
RVESVI: Right ventricular end-systolic volume index
RVMI: Right ventricular myocardial mass index
RVRI: Right ventricular remodeling index
